# Public views on a wait time management initiative: a matter of communication

**DOI:** 10.1186/1472-6963-10-228

**Published:** 2010-08-05

**Authors:** Rebecca A Bruni, Andreas Laupacis, Wendy Levinson, Douglas K Martin

**Affiliations:** 1 Joint Centre for Bioethics, University of Toronto, Toronto, Canada; 2 Centre for Clinical Ethics, Toronto, Canada; 3 Department of Health Policy, Management and Evaluation, University of Toronto, Toronto, Canada; 4 Keenan Research Centre, Li Ka Shing Knowledge Institute of St. Michael's Hospital, Toronto, Canada; 5 Department of Medicine, University of Toronto, Toronto, Canada

## Abstract

**Background:**

Many countries have tried to reduce waiting times for health care through formal wait time reduction strategies. Our paper describes views of members of the public about a wait time management initiative - the Ontario Wait Time Strategy (OWTS) (Canada). Scholars and governmental reports have advocated for increased public involvement in wait time management. We provide empirically derived recommendations for public engagement in a wait time management initiative.

**Methods:**

Two qualitative studies: 1) an analysis of all emails sent by the public to the (OWTS) email address; and 2) in-depth interviews with members of the Ontario public.

**Results:**

Email correspondents and interview participants supported the intent of the OWTS. However they wanted more information about the Strategy and its actions. Interview participants did not feel they were sufficiently made aware of the Strategy and email correspondents requested additional information beyond what was offered on the Strategy's website. Moreover, the email correspondents believed that some of the information that was provided on the Strategy's website and through the media was inaccurate, misleading, and even dishonest. Interview participants strongly supported public involvement in the OWTS priority setting.

**Conclusions:**

Findings suggest the public wanted increased communication from and with the OWTS. Effective communication can facilitate successful public engagement, and in turn fair and legitimate priority setting. Based on the study's findings we developed concrete recommendations for improving public involvement in wait time management.

## Background

Wait times have been ranked as a significant failing of public health systems in opinion surveys across several industrialized countries [1]. Waiting for care can lead to patient suffering, strained doctor-patient relationships and significant patient dissatisfaction [2]. However, there is no agreement on how to set wait time targets and prioritize wait lists.

Wait time management has been studied in many contexts, such as radiation oncology,[3] critical care,[4] intensive care,[5] limb arthroplasty,[6] emergency department,[7] and surgery[8]. Many countries have tried to reduce wait times through formal wait time reduction strategies [9,10]. Despite the vast array of wait time management efforts, the public (please see Table 1 for Definitions of Key Terms) have been involved in only a few [11].

**Table 1:** Definitions of Key Terms

**Public engagement**: The practice of involving members of the public in the agenda-setting, decision-making and policy-forming activities of a wait time management initiative.
**Public: **Citizens other than those affiliated with government and health care providers, employees of pharmaceutical and device companies, employees of disease-focused groups and elected officials.

For example, in June 2004, the National Health Service in the UK announced a wait time reduction effort, the "18 week patient pathway", which guarantees that no citizen would wait more than 18 weeks for surgery by 2008 [10]. The NHS's Patient and Public Champion Lead is responsible for ensuring the public experience is at the heart of the pathway's work, but they have not included lay representatives on decision making bodies to facilitate shared decision making [10]. A wait time reduction effort in New Zealand set priority criteria to reduce wait times in five areas of the health system and public forums were held to discuss the role of social factors in wait list prioritization [9]. However, the public were not consulted in the initial development of the criteria, nor were the public involved in any advisory committees developing priority setting criteria. These examples demonstrate that, even in contexts where the public have in some way been consulted, they have not been effectively involved in wait time management.

Many wait time initiatives have promoted their website as a vehicle for public involvement, and these websites have disseminated a wide range of information to the public [12,13]. For example, The New South Wales Health Department (Australia) wait time website allowed patients and physicians to search wait times for various procedures [14]. New Zealand's website for elective surgery provides information to the public about the booking system and clinical priority guidelines [15]. The UK's 18 Week Pathway website provided information on the strategy's goals and actions, and data on current wait times [10]. Cromwell *et al. *reviewed the websites of 6 government wait time initiatives and found that the wait time statistics were highly questionable because of the different types of data and aggregations employed; none of the websites stated whether the wait time statistics could be used to predict an expected waiting time; and most sites provided inadequate advice on how to appropriately interpret the information on the website [16].

### Increasing Public Involvement in Wait Time Initiatives

Scholars and governmental reports have advocated for increased public involvement in wait time management, and increased communication to provide information about the priority setting process and rationales to the public [17,18]. They argue that the fairness of wait time initiatives can be improved by involving all stakeholders and considering all relevant values - including the public [18]. The public wants to be consulted and educated about wait time management decision making [19]. They also want more transparent priority setting and more information about the priority scores used for waiting time management [20]. Such information may allow them to more readily accept waiting for care, and better equip them to deal with wait times [7,8].

Canada's Federal Advisor on Wait Times proposed that the government should disseminate information about actions provinces are taking to reduce wait times and why they are taking such actions, and that the public should be involved in the development of the education efforts to determine how to frame and disseminate the message to effectively reach Canadians [17].

### The Ontario Wait Time Strategy

The Ontario Wait Time Strategy (OWTS) (Canada) is a province-wide initiative to improve access and reduce wait times in five areas [21]. Previously we conducted a qualitative case study to describe and evaluate the priority setting activities of the OWTS, with particular attention to public engagement [22]. This previous study was guided by an explicit conceptual framework - 'accountability for reasonableness' [23] is a conceptual framework for legitimate and fair priority setting. It has gained international recognition and emerged as the leading conceptual framework for priority setting researchers [24]. To describe the priority setting process of the OWTS we used qualitative case study methods. There were two sources of data: (1) over 25 documents (e.g. strategic planning reports), and (2) 28 one-on-one interviews with informants (e.g. OWTS participants). Data was analyzed using a modified thematic technique in three phases: open coding, axial coding, and evaluation. Evaluation involved comparison between the description of the case study (i.e. what they did) with the conceptual framework, (i.e. what they should do). Points of agreement with the framework were considered good practice; points of divergence were marked as areas for improvement. The OWTS partially met the four conditions of 'accountability for reasonableness'. Study participants identified both benefits (i.e. experts of the lived experience) and concerns (i.e. public's lack of interest to be involved) for public involvement in the OWTS [22].

Additionally, in the previous study we found that there was no public involvement in the decisions of the OWTS, and that their website was the sole vehicle for public engagement. We found that the OWTS provided an email address on its website for the public to submit comments and questions, but the emails received by the OWTS were unanalyzed.

To our knowledge, no studies have described the views of members of the public about a specific wait time initiative. To fill this gap we conducted two qualitative studies: 1) an analysis of all emails sent by the public to the OWTS email address; and 2) in-depth interviews regarding the priority setting activities of the OWTS with members of the Ontario public. We provide empirically derived recommendations for public engagement in a wait time management initiative.

## Methods

The methods for the two studies will be presented separately. The methodology of both studies were guided by qualitative research methods, defined by Strauss and Corbin: "a nonmathematical process of interpretation, carried out for the purpose of discovering concepts and relationships in raw data and then organizing these into a theoretical explanatory scheme" [25].

### Methods - Analysis of Emails

#### Setting

As a result of the National Waiting Times Reduction Strategy, on November 17, 2004 the Ontario Ministry of Health and Long-Term Care introduced The Ontario Wait Times Strategy (OWTS), a province-wide initiative, to improve access, reduce wait times, set appropriate wait time targets, and develop a system to prioritize patients by need. Guided by the federal selection of the five wait time areas, the OWTS decided to specifically target: cancer surgery, cardiac revascularization procedures (coronary angiography, percutaneous coronary intervention, and coronary artery bypass graft surgery), cataract surgery, total joint hip and knee replacements, as well as Magnetic Resonance Imaging (MRI) and Computerized Tomography (CT) scans [21]. Since the inception of the OWTS $986 million (CAD) has been invested in wait time reduction efforts [26]. Funding for the OWTS is a joint venture from both the federal and provincial governments.

The OWTS launched a public website http://www.ontariowaittimes.com in December 2004. The website provided two portals: public and healthcare provider. The public website reported on the actions and plans of the strategy by posting formal reports (i.e. "Wait Time Updates") which provided a synopsis of the strategy's actions. The website provided general information on wait times including how wait lists are determined, and how long one can expect to wait. The public could search wait times according to procedures in varying geographic locations across the province, and provide comments via an email address.

#### Subject Sampling

All emails sent to the email address on the OWTS website, from the launch of the website in December 2005 until September 2007, were included in the email data set.

#### Data Collection

The emails were obtained from the MoHLTC in electronic form, in September 2007. The data set consisted of 116 emails, two of which were duplicates. In total, 114 emails were analyzed. Self-identified senders of the emails included the general lay public (83.6%), health care professionals (8.6%), and others (i.e. non-governmental associations, hospital CEOs, Members of Provincial Parliament, etc.). No other socio-demographic information was collected. The average length of the emails was 297 words.

#### Data Analysis

The data analysis proceeded in three phases: open, axial, and selective coding. First, in open coding, the data were read for familiarization and then re-read, examined and fractured into chunks of data that related to a concept or idea. Second, in axial coding, the concepts were organized into emerging themes that were derived from the data. The data set was re-read to allow comparison within and between the emails. Third, in selective coding, emerging themes were developed and illustrated through verbatim quotes from the data. These themes were derived based on the frequency and emphasis of their appearance in the data set.

We addressed the validity of the interpretations in three ways. First, two researchers (RAB and DKM) coded the raw data to ensure consistency and accuracy. Second, the emerging findings were presented to an interdisciplinary research team for questioning. This served to enhance 'reflexivity' (ensuring that prior assumptions, experience and personal bias were acknowledged), [27] and check preconceived assumptions. Third, a rigorous record of the data analysis and methodology was documented by the researcher (RAB) to allow for a critical appraisal of the methodology by the research team.

#### Research Ethics

Research ethics approval was granted from the Sunnybrook Health Sciences Centre and University of Toronto Research Ethics Board. A confidentiality agreement, written by the Ministry of Health and Long-Term Care, was binding to the researchers. All data and participation were kept strictly confidential and available exclusively to the research team. In dissemination of the research in any form, participants' anonymity has been strictly protected.

### Methods - Interviews

#### Setting

The St. Michael's Hospital Family Practice Unit is located in downtown Toronto, Canada. It is the Department of Family and Community Medicine's busiest outpatient clinic with approximately 44,000 visits per year. Its' catchment population includes a large portion of under-housed, recent immigrants, and thus looks after many patients of lower socioeconomic status.

#### Subject Sampling

Participants in the interviews were enrolled concurrently with the data analysis and new participants continued to be enrolled until the same views were heard repeatedly in consecutive interviews - sometimes called saturation[25]. Two sampling methods were used: convenience [25] and snowball sampling[28].

Participants were eligible if they were residents of Ontario, over the age of 18, and able to participate in a 20-minute interview in English. Healthcare workers, employees or directors of pharmaceutical companies and members of provincial parliament or other elected officials were excluded from study participation. Participants were recruited at the Family Practice Unit in two ways: (1) in-person, real time, recruitment in the clinic's waiting room area, and (2) posters and information sheets displayed in the clinic's waiting room area. Those individuals who chose to participate were asked to tell others about the study. A total of 34 individuals were recruited: 29 from in-person recruitment; 4 from study posters; and 1 from a referal by a previous participant.

Approximately 115 individuals were approached. The reasons people gave for declining the interview included: "don't have time", "not interested", or "can't speak English". The 34 interview participants were of varying backgrounds, including 18% immigrants, 65% female, 35% had a chronic health condition (e.g. diabetes, depression). Participants had a mean age of 51, with a range of 24 to 83 years.

#### Data Collection

The 34 interviews were conducted between August and September 2007 and were audio-taped and transcribed. An interview guide was developed based on previous research [22,29-31] and was revised during data collection in order to pursue emerging findings. For example, participants unexpectedly discussed distrust with the OWTS information. The interview guide was modified in order to pursue this concept. All participants were given a brief description of the purpose and activities of the OWTS prior to commencing the interview.

#### Data Analysis

The data analysis and methods of addressing the validity of the interpretations were the same as the analysis of emails.

#### Research Ethics

Research ethics approval was granted from the St. Michael's Hospital Research Ethics Board and the University of Toronto Health Sciences Research Ethics Board. All data and participation were kept strictly confidential and available exclusively to the research team. In dissemination of the research in any form, participants' anonymity has been strictly protected.

## Results

The results of the analysis of emails and the patient interviews are presented separately. Direct quotations are provided to illustrate the results. To avoid identifying specific individuals the participants have been allocated identification codes.

### Analysis of emails

#### Support for the Initiative

Five (of 116) email correspondents expressed support for the OWTS and described the OWTS as a worthwhile initiative because they believed that Ontarians are generally waiting too long for care. They suggested wait time reduction efforts would improve the health system.

I do believe in the wait time project and applaud this initiative... it can only improve health care. (Lay Public, 1)

#### Disappointment with the OWTS Communication Strategy

Overwhelmingly, email correspondents were dissatisfied with the communication efforts of the OWTS, specifically the OWTS's response to their emails and the information available on the OWTS website. Email correspondents wanted more communication with and information from the OWTS - they wanted to use the website as a vehicle to engage with the OWTS about the Strategy. As well, email correspondents doubted the legitimacy of information disseminated by the OWTS.

##### OWTS Responses

Ten email correspondents sent emails addressing a lack of response from the OWTS to their previous email(s). In addition, those who received a generic form-letter perceived it as insincere, and they expressed anger over the lack of a personalized response addressing their particular issue. Email correspondents wanted to be able to use the OWTS email address as a method to engage with the OWTS about their questions and concerns, but their emails were either not acknowledged or acknowledged only generically.

What must be done to get you people to reply to e-mails that are sent to you? This is my third follow-up to my original message!! You should remove the capacity for persons to contact you by e-mail if, as appears to be the case, your policy is just to ignore e-mail messages that people send you. (Lay Public, 2)

##### The OWTS website

Twenty four email correspondents requested additional information beyond what was offered on the OWTS website. Examples of information requests included clarification of information, directions for how to move from a current wait list to a shorter wait list, and requests for wait time statistics not available on the website.

What are my next steps to get an MRI in a reasonable time frame - not 224 days that is current wait list in Kitchener, Ontario? (Lay Public, 3)

#### Distrust of OWTS Information

##### Wait Time Statistics

Sixty-two email correspondents shared angry sentiments about the Strategy's wait time statistics. They believed that statistics disseminated to the public via newspapers, the television, and the website were false. They articulated a desire for truthful information, even if it was discouraging.

I was told he would book the [cataract] surgery but was already booking into March/07. This does not line up with the stories in the local papers that wait time for cataract surgery is 128 days. How can anyone believe anything they are told or read? I believe the Ministry is not being honest about wait times ...Why is the reality so different than the government's stories about shortened wait times? (Lay Public, 4)

##### Television Advertisements

Seventeen email correspondents shared angry sentiments about the OWTS television advertisements. They asserted the advertisements were a waste of resources and misleading to the public - they gave a message that all wait times were being reduced, when only five areas of care were being targeted. It was suggested the resources spent on the commercials should be spent directly on wait time reduction efforts.

I find your recent TV commercial for wait times very misleading and as such, if I were you, I'd be very embarrassed to even put it out there. You give the impression that the wait times for ALL doctors have decreased, which is NOT the cases whatsoever....As a Ontarian I am quite embarrassed to see those ads and feel you should remove them immediately and put some TRUTH in those ads before running them again. (Health Care Worker)

##### Wait Time Definition

Twelve email correspondents claimed that the definition of wait times is misleading and contributes to the deceitfulness of the wait time statistics as it does not account for the time waited to see a general practitioner or a specialist.

I have found that the wait time statistics are flawed! ...This may be correct if only counting the time a person has to wait from the time he/she sees the surgeon to schedule surgery and the time surgery is performed.... In other words your statistics are flawed and give an incorrect picture of health care in Ontario. (Lay Public, 5)

### Part Two - Interview Findings

#### Participants' Views on Public Involvement in the OWTS

All 34 interviewees strongly supported public involvement in the OWTS priority setting, describing it as a necessary component of decision making.

I think anything to do with the Government -- [for instance] when it comes to introducing more funding, more staff into the healthcare system -- I think the public should always be involved. (Male Patient, A)

Most interviewees suggested that members of the public can offer insight into the lived experience of being ill and using the health system. Such a perspective cannot be offered by 'experts' and is relevant to priority setting.

We're the ones that experience the healthcare system. To me, the politicians are sitting behind a desk. They're not sitting in emergency waiting. They're not sitting with their dying parent. Now, I'm sure they've had those experiences, but I think the public in general should be in on making decisions as to how our healthcare system goes. (Female Patient, A)

According to participants, members of the public can best identify the needs of the community.

Well, it's the public that's experiencing wait times. If there isn't any input from the public, like in business if there isn't any input from the customer, how do you really identify what the needs are? (Male Patient with Chronic Health Condition, B)

##### Enhanced 'Buy-in'

Seven participants proposed that the public will be more likely to buy into the initiative and support the actions and priority setting decisions of the initiative if they are involved.

Because then you get buy-in with the whole process. I think [public involvement] would help [the OWTS] because [the public] are not feeling that they have any control over the health system. You just don't feel like you're part of it, and if you're not part of it you're against it. Like if having the public come in and at least getting their opinion, then you get some sort of buy-in. (Female Patient with Chronic Health Condition, B)

##### Shared Decision Making

Seventeen participants supported shared decision making between experts and the public. They described an auxiliary role for the public in priority setting decision making - the public should collaborate with experts in the decision making process.

So it would be nice if the Government before spending money could talk about their plans for the public, so the public can share their decision with them. (Male Patient, A)

#### Disappointment with the OWTS Communication Strategy

Interview participants did not feel they were sufficiently made aware of the OWTS or its' actions. Less than half of the group interviewed was aware of the OWTS (47%); most became aware of the Strategy via the radio or television. Less than one fifth (15%) of those interviewed were familiar with the details of the Strategy (e.g. Which health care areas were prioritized by the Strategy? What efforts the province had taken to reduce wait times?). Only one interview participant was aware of the OWTS website.

Eleven participants expressed concern that the public were not well enough informed of the activities of the OWTS. To facilitate effective public involvement, participants suggested the OWTS needs to communicate more information to the public about the Strategy. The public should be informed of the Strategy's activities and relevant issues prior to decision-making.

They aren't communicating very well with the public for us to be informed to make decisions anyways. If we don't have ...information [about the OWTS], then we can't truly make a decision as a public whole what our needs are. I just find, they communicate what they want us to hear. (Female Patient, C)

Two participants suggested lack of communication about the OWTS to the public can lead the public to misinterpret the purpose of the strategy.

I think there's a whole lot of, I don't know if it's misinformation, but people should be able to inform themselves or someone should inform them what [the OWTS is] all about. ...it's just a matter of education. (Male Patient, C)

Two participants proposed that if the public were given more information on wait times they would be more willing to wait for care, as long as the wait time is within medically acceptable standards.

I do think that if the person is knowledgeable about the risk of waiting, and that would be important to communicate that to the public -- because I don't mind waiting six months if I know it is for a routine test.....I am trusting the government to know that that is an acceptable standard. (Male Patient, D)

## Discussion

Findings suggest the public want increased communication from and with the OWTS. Effective communication with the public can facilitate successful public engagement, and in turn fair and legitimate priority setting. To our knowledge, this is the first study that has described the views of members of the public about a wait time management initiative, with a specific focus on public engagement. These findings will be helpful to the leadership of the OWTS and could be helpful to leaders of wait time initiatives elsewhere.

We found that members of the public wanted to be more informed about the OWTS and its actions, and wanted the public to participate in the priority setting of the OWTS. Previous research has similarly reported that the public want to be better informed of the actions of wait time management initiatives and desired to participate in decision making [19,20].

The key findings from our study concerned the provision of information by the OWTS. Although the OWTS's website was intended to disseminate information to the public, our participants were not satisfied with the information provided by the OWTS -- they wanted more information. Moreover, the members of the public in our study believed that some of the information that was provided by the OWTS -- on its website and through the media -- was inaccurate, misleading and even dishonest. Patients in other wait time studies have suggested receiving accurate information on wait times and reasons for waiting will help them to better deal with waiting for care [7,8].

Most participants of the interview study were not aware of the OWTS' efforts to disseminate information about the strategy through their website, media briefings, and television advertisements, which raises questions both about the effectiveness of these communication strategies and the public's willingness to spend time informing themselves about the OWTS. Some email correspondents who were aware of these efforts were angered by the OWTS television advertisements and suggested the advertisements were a waste of money. The OWTS needs to reconcile this dichotomy - that some members of the public want more communication, while others were angered by certain communication efforts, particularly the television advertisement.

The OWTS website clearly stated that the OWTS's definition of wait time was the time from the decision that surgery was indicated to the time of surgery, and did not claim to incorporate the time waited to see a general practitioner and a specialist. However, many participants in our study distrusted the OWTS website because this definition did not correspond with their experience of waiting for care, which includes time waiting to see a primary care physician and the time waiting to see a specialist. Consequently, participants believed the wait time statistics were conceptually flawed and biased toward being short, and this increased their skepticism about the entire OWTS. This level of skepticism might have been decreased if the OWTS website explicitly acknowledged that the strategy was at present only focusing on one aspect of the wait times experienced by patients, and that the other wait times (e.g. waiting to see a family physician, waiting to see a surgeon) are extremely important as well.

Some study participants distrusted the OWTS because their own wait times were longer than the wait times reported on the website. This suggests that the some members of the public do not know how to interpret wait time statistics, which invariably represent a summary of wait times (e.g an average wait time, or the maximum length of time waited by 90% of patients). It seems unfair to blame the OWTS for this, but it does suggest that more effort needs to be spent explaining how to interpret the figures presented on the website, and to explicitly indicate that some patients will wait longer than the numbers suggest.

Based on the findings from this study, our previous study of the OWTS [22], current public involvement literature, and the 'accountability for reasonableness' framework we suggest concrete ways for improving public engagement in wait time management in Table 2: Recommendations for Public Engagement in the Ontario Wait Time Strategy. The recommendations include: *1) Shared Decision-Making *- collaboration between the public and 'experts' will enhance legitimacy and fairness at all stages of OWTS decision making. Both participants from this and the previous study supported public involvement in decision making, and suggested the public participate as shared decision-makers. Participants suggested creating public positions on the expert panels as a way to facilitate public participation. Additionally participants of the previous study and of the interviews suggested creating a public committee as a vehicle to facilitate ongoing public consultation (i.e. a citizens' council). 2) *Communication Strategy *- enhanced communication will facilitate effective public engagement, and in turn fair and legitimate priority setting. Both email correspondents and interview participants wanted more communication with and information from the OWTS. Findings from our previous study identified poor communication with the public about the OWTS an area in need of improvement. 3) *Feedback and Appeals Mechanism *- a formal mechanism, with channels to decision makers, to permit public feedback on priority setting activities will enhance the responsiveness of the strategy and the legitimacy and fairness of priority setting. The previous study of the OWTS found that there was no formal feedback and appeals mechanism for stakeholders on OWTS priority setting. Additionally, the email correspondents tried to use the OWTS email address as an informal feedback and appeals mechanism, but were unsuccessful.

**Table 2:** Recommendations for Public Engagement in the Ontario Wait Time Strategy

*Foci*	*Operational Plan*
**Shared Decision Making**	1) Create positions for public members on expert panels.
	create two public positions on each expert panel to help mitigate the potential power differences between 'experts' and the public
	provide training workshops to educate the members about the initiative, and on their roles and responsibilities for participating on the panel
	2) Construct a Citizens' Council, consisting of the assembled public from the expert panels, to collaborate with the OWTS and provide ongoing advice on priority setting.
	engage the public in developing a definition of wait times that corresponds to patients' lived experience, identifying criteria that will serve as a guide to priority setting in general, and the selection of future target service areas
	advice of the Citizens' Council can be incorporated with that of other stakeholders

**Communication Strategy**	1) Create a communication panel, including expert and public members, to develop an effective communication strategy aimed at all stakeholders, especially the public.
	disseminate the actions of the strategy, and the rationales (how? and why?) used
	public members can advise the communication on what information about the strategy the public would like disseminated, and effective vehicles for message-framing
	efforts should be made to better design the website so that the public is not disenfranchised by misinterpreting information on the website

**Feedback & Appeals Mechanism**	1) Establish a formal feedback/appeals mechanism for all stakeholders, including the public.
	create a feedback section on the OWTS website - provide established questions about OWTS priority setting (e.g. What areas of care would you like the OWTS to include if its' priority areas are expanded?)
	conduct a series of randomly distributed mail out questionnaires to the public to obtain their views on the priority setting
	2) Synthesize and analyze the feedback
	both the OWTS leaders and the citizens' council should periodically provide a public report on the feedback providing: a summary of the feedback, and the corresponding action(s) taken to address the key issues identified from the feedback

Implementing an extensive public engagement strategy at the OWTS raises some important questions: Do all wait list initiatives need an extensive public engagement strategy? Should similar public engagement strategies be instituted in other contexts of the health system (i.e. nursing homes, hospitals)? Do we need Citizens' Councils for all areas of health care? There are insufficient resources to implement expansive public engagement strategies in all contexts of the health system and in every wait list management initiative. Public engagement efforts should be proportional to the importance of the initiative. Wait times initiatives are important -- according to public opinion surveys across several industrialized countries wait times are considered a significant failing of public health systems [1]. Even if extensive public engagement throughout an entire health system is not practical, decision makers should strive to implement some degree of public engagement - public engagement is not an all-or-none phenomenon. Further, the public can be involved in many ways ranging from didactic communication efforts to shared- decision making. Effective public engagement enhances the legitimacy and fairness of decision making, which is a key overarching goal of public policy making.

Is there not some responsibility of the public to be proactive in putting their views forward? The public have some responsibility in utilizing avenues available to them to voice their views, such as an email address set up for their feedback. However, this avenue may prove unfruitful if no one reviews, or responds to, their emails. Where no public engagement vehicles are provided, citizens often resort to public demonstrations that capture media attention, which may be embarrassing for a government but is usually less effective at stimulating policy change. An effective public engagement strategy sends a message to the public that their views are important. This in turn may increase public support of the initiative and trust in the decision makers.

## Limitations

The primary limitation of this study is that the findings may not be generalizable to all members of the public or to other wait list strategies. Each study group provided a limited perspective, in particular the emails correspondents. The email correspondents were likely more disgruntled than the average citizen and thus may not represent the views of Ontario citizens. It is also likely that seniors and individuals of a low socio-economic status are not as likely as the average Ontarian to be represented by the email correspondents' views. No socio-demographic information was collected on the email correspondents or on the average user of the OWTS website. However, the email correspondents' responses provide a relevant perspective. The views described from the interview study were limited to one stakeholder group: Ontarians living in Toronto who were visiting their general practitioner. However, generalizability was not a goal of this study. Each study provided a rich description and a valuable contribution to the knowledge base. It is likely that lessons from the studies will be helpful to others in wait list management and other priority setting contexts. Input from other groups of stakeholders would provide an ever richer description and is a potential for future research. Moreover, the methods can be duplicated with great benefits in other contexts. Second, this study is time limited, and the OWTS is an ongoing and dynamic initiative, which is continuing to learn and revise its strategy. However, the majority of key priority setting decisions pertaining to the OWTS have been made prior to and during this study period. The third limitation is social desirability bias -- interviewees' views may reflect what they thought the researchers wanted to hear. However, the parallel analysis of emails provides verification for the interview data.

## Conclusions

Communication with the public is an essential component of public engagement. Findings suggest the public wanted increased communication from and with the OWTS. Effective communication can facilitate successful public engagement, and in turn fair and legitimate priority setting. Empirically grounded recommendations for how to engage the public in a wait time management initiative have been developed based on prior research which provided an in-depth description and evaluation of the OWTS [22] and this study's examination of the views of members of the public on priority setting, public engagement and wait time management.

## Competing interests

The authors declare they have no competing interests.

## Authors' contributions

RB conducted the data collection (interviews and email analysis) on which this paper is based, collated and analyzed the data, and drafted the manuscript. DKM participated in analyzing the data and commented on earlier drafts of the manuscript and was involved in revising it critically for important intellectual content. AL commented on earlier drafts of the paper. WL commented on earlier drafts of the paper. All authors made substantial contributions to the conception and design of the study and read and approved the final manuscript.

## Pre-publication history

The pre-publication history for this paper can be accessed here:

http://www.biomedcentral.com/1472-6963/10/228/prepub

## References

[B1] Hurst J Siciliani L Tackling Excessive Wait Times for Elective Surgery: A comparison of policies in twelve OECD countries 2003 OECD Health Working Papers. Paris 10.1016/j.healthpol.2004.07.003 15802155

[B2] D'Souza DP Martin DK Purdy L Bezjak A Singer PA Waiting lists for radiation therapy: a case study BMC Health Serv Res 2001 1 11319944 10.1186/1472-6963-1-3 PMC32176

[B3] Palda VA Llewellyn-Thomas HA Mackenzie RG Pritchard KI Naylor CD Breast cancer patients' attitudes about rationing postlumpectomy radiation therapy: applicability of trade-off methods to policy-making J Clin Oncol 1997 15 3192 200 9336355 10.1200/JCO.1997.15.10.3192

[B4] Cooper AB Joglekar AS Gibson J Swota AH Martin D Communication of bed allocation decisions in a critical care unit and accountability for reasonableness BMC Health Serv Res 2005 5 67 10.1186/1472-6963-5-67 16259634 PMC1298296

[B5] Martin DK Singer PA Bernstein M Access to intensive care unit beds for neurosurgery patients: a qualitative case study J Neurol Neurosurg Psychiatry 2003 74 1299 303 10.1136/jnnp.74.9.1299 12933940 PMC1738671

[B6] Prasad S Kapoor PK Kumar A Reddy KT Kumar BN Waiting-list prioritization in the National Health Service J Laryngol Otol 2004 118 39 45 10.1258/002221504322731619 14979971

[B7] Cross E Goodacre S O'Cathain A Arnold J Rationing in the emergency department: the good, the bad, and the unacceptable Emerg Med J 2005 22 171 6 10.1136/emj.2004.020180 15735262 PMC1726698

[B8] Fitzsimons D Parahoo K Stringer M Waiting for coronary artery bypass surgery: a qualitative analysis J Adv Nurs 2000 32 1243 52 10.1046/j.1365-2648.2000.01595.x 11115010

[B9] Gauld R Derrett S Solving the surgical waiting list problem? New Zealand's 'booking system' Int J Health Plann Manage 2000 15 259 72 10.1002/hpm.596 11246897

[B10] 18 Week patient Pathway Website. Delivering the 18 week patient pathway 2008 National Institute of Health http://www.18weeks.nhs.uk/public/default.aspx?load=HomeNews

[B11] Kenny N Joffres C An ethical analysis of international health priority-setting Health Care Anal 2008 16 145 60 10.1007/s10728-007-0065-5 18449806

[B12] Glynn P Taylor M Hudson A Surgical Wait List Management: a strategy for Saskatchewan. A Report to Saskatchewan Health 2002 10.12927/hcq..16680 12055865

[B13] Wait times Health Care Renewal in Canada: Clearing the Road to Quality 2006 Toronto: Health Council of Canada

[B14] NSW Hospital Waiting Times Website 2007 New South Wales Department of Health http://www.health.nsw.gov.au/waitingtimes/dwt.html

[B15] Website for Elective Services. New Zealand Ministry of Health 2008 http://www.electiveservices.govt.nz/

[B16] Cromwell DA Griffiths DA Kreis IA Surgery dot.com: the quality of information disseminated by Web-based waiting time information services Med J Aust 2002 177 253 5 12197820 10.5694/j.1326-5377.2002.tb04758.x

[B17] Postl B Final Report of The Federal Advisor on Wait Times 2006 Ottawa: Health Canada

[B18] Conner-Spady B Estey A Arnett G Ness K McGurran J Bear R Noseworthy T Determinants of patient and surgeon perspectives on maximum acceptable waiting times for hip and knee arthroplasty J Health Serv Res Policy 2005 10 84 90 10.1258/1355819053559155 15871767

[B19] From Chaos to Order: Making Sense of Waiting Lists In Canada Final Report 2001 Edmonton: Western Canada Wait List Project

[B20] Edwards RT Boland A Wilkinson C Cohen D Williams J Clinical and lay preferences for the explicit prioritisation of elective waiting lists: survey evidence from Wales Health Policy 2003 63 229 37 10.1016/S0168-8510(02)00101-X 12595123

[B21] Ontario Wait Times Website. Ministry of Health and Long-Term Care 2007 http://www.health.gov.on.ca/en/public/programs/waittimes/

[B22] Bruni R Laupacis A Levinson W Martin DK Public involvement in the priority setting activities of a wait time management initiative: a qualitative case study BMC Health Serv Res 2007 7 186 10.1186/1472-6963-7-186 18021393 PMC2238747

[B23] Daniels N Sabin J Setting Limits Fairly: Can we learn to share medical resources? 2002 Oxford: University Press

[B24] Ham C Coulter A Global Challenges of Health Care Rationing London 2000 UK: Open University Press

[B25] Strauss A Corbin J Basics of Qualitative Research: techniques and procedures for developing grounded theory 1998 Thousand Oaks, CA: SAGE Publications

[B26] Trypuc J The Wait Time Strategy Review of Activities: Update #9. Wait Time Updates 2007 Toronto: Ontario Wait Time Strategy

[B27] Denzin N Lincoln Y Handbook of Qualitative Research 2000 Thousand Oaks, CA: Sage Publications

[B28] Polit DF Beck CT Hungler BP Essentials of Nursing Research: Methods, Appraisal, and Utilization 2001 Philadelphia: Lippincott

[B29] Kapiriri L Opportunities for public accountability in priority setting: the case of Uganda. Bergen: 2003 University of Bergen

[B30] Singer P Martin D Giacomini M Purdy L Priority setting for new technologies in medicine: qualitative case study British Medical Journal 2000 321 1316 9 10.1136/bmj.321.7272.1316 11090513 PMC27534

[B31] Walton NA Martin DK Peter EH Pringle DM Singer PA Priority setting and cardiac surgery: a qualitative case study Health Policy 2007 80 444 58 10.1016/j.healthpol.2006.05.004 16757057

